# A Class Practice Study of Intervention Effect of Interactive Assessment on Learning Goal Orientation

**DOI:** 10.3389/fpsyg.2021.599480

**Published:** 2021-08-12

**Authors:** Mai Yokoyama, Kazuhisa Miwa

**Affiliations:** ^1^Graduate School of Informatics, Nagoya University, Nagoya, Japan; ^2^Center for Teaching and Learning, Teikyo University, Hachioji, Japan

**Keywords:** learning goal orientation, performance goal orientation, self-assessment, peer-assessment, intervention

## Abstract

While focusing on the moderating effects of initial performance-approach goal orientation and performance-avoidance goal orientation, this study aimed to examine the effects of self- and peer-assessment on the growth of learning goal orientation. We set up a control group and two experimental groups (self-assessment and peer-assessment groups) and conducted experimental lessons. The responses of the 63 subjects (control group: *n* = 14; self-assessment group: *n* = 25; peer-assessment group: *n* = 24) who attended these lessons were analyzed. The following observations were made: (1) the effect of peer-assessment on the growth of learning goal orientation may change depending on the initial performance-approach goal orientation or performance-avoidance goal orientation; (2) to increase learning goal orientation for students who have high performance-approach goal orientation or low performance-avoidance goal orientation, peer-assessment is effective; and (3) to increase learning goal orientation for students who have low performance-approach goal orientation or high performance-avoidance goal orientation, peer-assessment appears to be counterproductive.

## Introduction

### Goal Orientation

Dweck (1986) proposed goal achievement theory, which explains the differences in learning behavior depending on students’ goals. Students’ goals are classified into two categories according to this theory: learning goals and performance goals. The aim of learning goals is to learn new skills and knowledge through challenging learning activities, while those of performance goals are to seek positive and avoid negative evaluations. Regardless of their confidence in their abilities, students who are oriented toward learning goals tend to select challenging tasks and remain motivated even when they fail. Performance goal-oriented students behave similarly to students who have a learning goal orientation, given their confidence in their abilities; however, if they are not confident in their abilities, they do not continue to stay motivated and will apply passive strategies to complete tasks.

Many studies have investigated the relationship between goal orientation measured by questionnaires and variables in academic achievement, including Ames and Archer (1987, 1988). Kaplan and Midgley (1997) observed that learning goal orientation positively affects adaptive learning behaviors, and that performance goal orientation has no relationship to or negative effects on adaptive learning behaviors. Nolen and Haladyna (1990) demonstrated that learning goal orientation positively affects deep-processing behaviors, such as elaborating ideas and monitoring comprehension and memory. Similar results were reported by Fenollar et al. (2007) and Liem et al. (2008), and learning goal orientation has also been shown to predict motivational variables, such as intrinsic motivation (e.g., Heyman and Dweck, 1992; Kavussanu and Harnisch, 2000). The superiority of learning goal orientation has consistently been positively related to adaptive learning in recent studies (e.g., Chea and Shumow, 2014; Hudaykulov et al., 2015; Tercanlioglu and Demiröz, 2015).

Performance goal orientation was divided into performance-approach goal orientation, wherein a student attempts to outperform others, and performance-avoidance goal orientation—the desire to avoid performing worse than others (Elliot and Harackiewicz, 1996). Elliot and colleagues have observed that performance-approach goal orientation results in positive effects on various variables in academic achievement, such as endogenous motivation and academic performance, whereas performance-avoidance goal orientation has negative effects on these (e.g., Elliot and Church, 1997; Elliot and McGregor, 1999; Elliot et al., 1999; Rawsthorne and Elliot, 1999). Subsequent studies have shown results consistent with the findings of Elliot and colleagues (e.g., Chen and Wong, 2014; Li and Shieh, 2016; Nasiri et al., 2017), demonstrating the importance of distinguishing between performance-approach goal orientation and performance-avoidance goal orientation.

Can educational intervention enhance learning goal orientation, which has consistently emphasized superiority? Previous studies have shown that students in classes that see success as an improvement and focus on the learning process tend to be oriented toward learning goals, while students in classes that see success as good grades and focus on performance compared to other students tend to be oriented toward performance goals (Roeser et al., 1996; Anderman and Anderman, 1999). In other words, students’ learning goal orientation changes depending on the learning environment. Furthermore, Manalo et al. (2018) showed how the learning environment is designed and how teachers engage with students determine whether students’ spontaneity is promoted. From this finding, it is considered that students’ learning goal orientation may be enhanced by educational intervention.

To create a classroom environment that increases students’ learning goal orientation, Ames (1992) proposed instructional strategies from three dimensions: “task,” “authority,” and “evaluation/cognition.” The proposal emphasizes providing opportunities for students to participate in decision-making and individual students to grow and develop. However, we can find no empirical study that has investigated this proposal. Geitz et al. (2015) attempted to increase students’ learning goal orientation by increasing student involvement in their assessment and examined the effects on learning goal orientation. However, this method did not have a direct influence on the learning goal orientation. An effective intervention method and related knowledge to increase learning goal orientation have not yet been established; therefore, this study clarifies basic knowledge about interventions to increase learning goal orientation.

### Intervention on How to Assess: Self-Assessment/Peer-Assessment

In this study, we focused on assessing interventions to increase learning goal orientation. Assessment has a critical influence on student learning, increasing effort, motivation, and engagement (Kluger and DeNisi, 1996; Hattie and Timperley, 2007).

Self-assessment, in which students assess their own performance, and peer-assessment, in which students assess each other’s performances, are effective assessment methods. According to Adachi et al. (2018), when teachers assess students, beginners often feel passively criticized by experts. Self- and peer-assessment can reverse this relationship, thereby empowering students to be active assessors and emerging experts; similar opinions have been reported in many previous studies (e.g., Thomas et al., 2011; Sambell, 2016).

Geitz et al. (2015) explained that assessments that encourage students to be actively involved in their learning and be agents of their own change might effectively stimulate their learning goal orientation. Based on the above, we expected that self- and peer-assessment might increase learning goal orientation. Therefore, in this study, we examined the effects of self- and peer-assessment as interventions on eliciting the growth of learning goal orientation.

### Moderating Effect of Initial Performance Goal Orientation

Intervention does not work for all students; it depends on students’ beliefs and other characteristics (Bell and Kozlowski, 2002). What is important for an effective assessment is how students perceive the assessment and how it is received; how the assessment is felt and received is related to their goal orientation (Evans, 2013). Performance-approach goal orientation and performance-avoidance goal orientation are orientations related to assessment from others and, thus, are considered to be closely related to interventions on how to assess, the focus of this study. Therefore, the effects of the intervention on how to assess may vary depending on the goal orientation. For example, peer-assessment is expected to have an opposite effect on and decrease the learning goal orientation of students with high performance-avoidance goal orientation, who do not want to be assessed badly by others. Therefore, we focused on the moderating effects of initial performance goal orientation.

### Purpose

**FIGURE 1:**
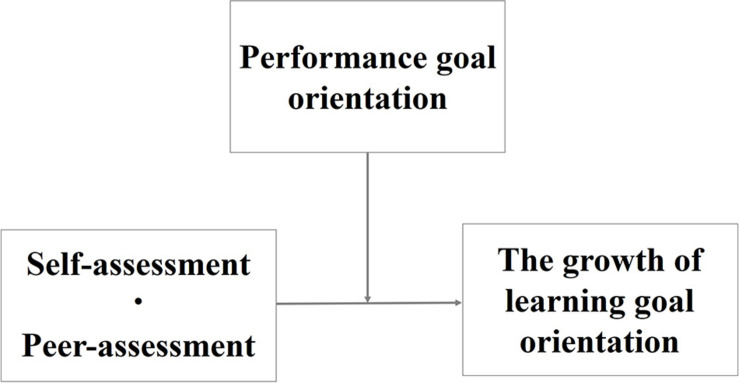
Outline of the study.

An outline of this study is presented in Figure 1. The purpose of this study was to examine the effects of self- and peer-assessment on the growth of learning goal orientation, focusing on the moderating effects of initial performance goal orientations. We examined the interaction effects of self-assessment/peer-assessment and initial performance goal orientations on the growth of learning goal orientation; we expressly organized a control group and two experimental groups (self-assessment and peer-assessment groups) and conducted experimental lessons.

## Materials and Methods

This experiment was conducted in a first-year class held at a Japanese public university. The experiment was carried out five times every other week from May to June 2018. The learning topic was debate training. Participants were 67 students and randomly divided into the control group (*n* = 14), self-assessment group (*n* = 27), and peer-assessment group (*n* = 26). Each group was subsequently divided into smaller groups (three to five students per group) and worked on group learning. The class procedure is shown in Table 1. The first author provided explanations and instructions in all classes, and the other five teachers acted as facilitators to support smooth debate practice and assessment activities.

**TABLE 1:** Class procedure.

Week 1	• Pre-test • Confirmation of class goals • Lecture on the basics of debate
Week 2	• Practice for debate
Week 3	• Preparation and practice for the match up debate
Week 4	• Preparation and practice for the match up debate • Intervention on how to assess (Self-assessment/Peer-assessment)
Week 5	• Match up debate • Post-test

We explained to the students that participating in this study was voluntary and that there was no disadvantage if they did not agree to participate. All the students participated in this study. Since this study was conducted in class, no reward was paid. As with the experimental group, the students assigned to the control group were given the experience of learning to assess using a rubric after the experiment. In doing this, control students were not at any educational disadvantage.

### Pre- and Post-test

We conducted a questionnaire survey on goal orientation initially in the course (pre-test) and eventually (post-test). Goal orientation was measured using 18 items from the Achievement Goal Scale developed by Elliot and Church (1997). Each item is rated on a five-point scale. This scale consists of the following three factors: learning goal orientation (e.g., “I want to learn as much as possible from classes.”); performance-approach goal orientation (e.g., “It is important to me to do better than the other students.”); and performance-avoidance goal orientation (e.g., “I worry about the possibility of getting a bad grade.”). We explained to the students that the questionnaire responses were not related to grades and that all responses were statistically analyzed so that the anonymity of the respondents was guaranteed.

### Intervention on How to Assess (Self-Assessment/Peer-Assessment)

When practicing debate, students in the self-assessment group assessed their own performance, and students in the peer-assessment group assessed students in other groups. Students in the experimental groups were informed that the assessment aimed to bridge the gap between the actual level of performance and the desired learning goal. Using a debate rubric, students in the experimental groups assessed and wrote about what they did well, what they should improve, and how they could improve their performance. As an example of the contents of the rubric, when the evaluation viewpoint of the rubric is “Logically compose the story,” the evaluation criteria were as follows: Level 0: The story is not logical, and the claim is difficult to understand; Level 1: Sometimes the story is illogical, and the claim is confusing; Level 2: The story is almost logically structured, giving examples and evidence; and Level 3: The story is logical and persuasive, giving examples and evidence. To verify the effect of assessing using a rubric created by the students themselves, half of the students in the experimental groups used a rubric created by a teacher, and the other half used a rubric created by themselves. The rubric created by the teacher was created by the first author. Students in the control group only practiced debate and did not assess their performance during this time.

## Results

The responses of the 63 subjects (control group: *n* = 14; self-assessment group: *n* = 25; peer-assessment group: *n* = 24) who answered all questions in the questionnaire and attended all classes were analyzed.

### Scale Structure

We conducted a confirmatory factor analysis to confirm the three-factor structure: learning goal orientation, performance-approach goal orientation, and performance-avoidance goal orientation. The fitness of the factor structure was evaluated based on the values of Comparative Fit Index and Root Mean Square Error of Approximation. Values above 0.95 for Comparative Fit Index and below 0.07 for Root Mean Square Error of Approximation were regarded as indicating sufficient fit (Hooper et al., 2008). After excluding three items that had a factor loading of 0.40 or less, the aforementioned criterion was satisfied: Comparative Fit Index-pre = 0.963, Root Mean Square Error of Approximation-pre = 0.047, Comparative Fit Index-post = 0.955, Root Mean Square Error of Approximation-post = 0.061. Hence, we concluded that the three-factor structure was acceptable. The Cronbach’s α coefficient for the pre- and post-test were 0.71 and 0.77 for learning goal orientation; 0.85 and 0.86 for performance-approach goal orientation; and 0.73 and 0.78 for performance-avoidance goal orientation. The average value of the four items was regarded as the learning goal orientation. The average value of the six items was regarded as the performance-approach goal orientation. The average value of the five items was regarded as the performance-avoidance goal orientation. The fundamental statistics for each variable are listed in Table 2.

**TABLE 2:** The fundamental statistics.

		All (*n* = 63)		Control Group (*n* = 14)		Self-assessment group (*n* = 25)		Peer-assessment group (*n* = 26)	
		*M*	SD	*M*	SD	*M*	SD	*M*	SD
Learning goal Orientation	Pre	3.92	–0.57	3.82	0.71	3.84	0.60	4.07	0.43
	Post	4.03	0.59	3.91	0.77	3.96	0.58	4.17	0.48
Performance-approach goal orientation	Pre	3.35	0.71	3.30	0.76	3.29	0.73	3.44	0.69
	Post	3.25	0.71	3.14	0.80	3.24	0.73	3.31	0.66
Performance- avoidance goal orientation	Pre	3.81	0.63	3.90	0.64	3.69	0.68	3.88	0.57
	Post	3.70	0.71	3.54	0.84	3.68	0.81	3.81	0.50

### Goal Orientation Before the Intervention

A one-way ANOVA was performed with groups as the independent variable and initial three goal orientation as the dependent variables. There was no significant difference in any of the analyses [learning goal orientation-pre: *F*(2,60) = 1.31, *n.s*.; performance-approach goal orientation-pre: *F*(2,60) = 0.30, *n.s.*; performance-avoidance goal orientation-pre: *F*(2,60) = 0.77, *n.s.*]. This result confirmed that there was no difference in goal orientation among the three groups before the intervention.

### Effect on Growth of Learning Goal Orientation

By subtracting the learning goal orientation-pre from the learning goal orientation-post, the value obtained was used as the variable for the growth of learning goal orientation. We assigned 0, 1, and 0 to the control, self-assessment, and peer-assessment groups, respectively, as dummy variables expressing the effect of self-assessment (self-assessment dummy). We assigned 0, 0, and 1 to the control, self-assessment, and peer-assessment groups, respectively, as dummy variables expressing the effect of peer-assessment (peer-assessment dummy). The correlation between independent variables becomes high, and multicollinearity occurs when an interaction term is created using the raw score of the moderating variables; however, by subtracting the average value from the score of the moderating variable, this problem can be avoided (Aiken and West, 1991). Therefore, the values obtained by subtracting the average value of the entire sample from the raw scores of individual students were used as the performance-approach goal orientation-pre and performance-avoidance goal orientation-pre variables.

Hierarchical multiple regression analysis was performed with the growth of learning goal orientation as the dependent variable. Self-assessment dummy, peer-assessment dummy, performance-approach goal orientation-pre, and performance-avoidance goal orientation-pre were entered as the independent variables. The main effect of each variable was examined in Step 1. In Step 2, four interaction terms (self-assessment dummy × performance-approach goal orientation-pre, self-assessment dummy × performance-avoidance goal orientation-pre, peer-assessment dummy × performance-approach goal orientation-pre, and peer-assessment dummy × performance-avoidance goal orientation-pre) were entered as independent variables, and the moderating effects of performance-approach goal orientation and performance-avoidance goal orientation were examined.

The results are shown in Table 3. No main effect of any independent variable is shown in step 1. In Step 2, it was confirmed that the increment of the variance explanation rate was significant (Δ*R*^2^ = 0.21, < 0.05) and that the explanation rate of learning goal orientation increased when the interaction terms were entered.

**TABLE 3:** Results of hierarchical multiple regression analysis.

Independent variable	β	ΔR^2^
**Step 1: Main effect**		
Self-assessment dummy	0.02	0.02
Peer-assessment dummy	–0.01	
Performance-approach goal orientation-pre	0.14	
Performance-avoidance goal orientation-pre	–0.02	
**Step 2: Moderating effect**		
Self-assessment dummy × Performance-approach goal orientation-pre	0.19	21*
Self-assessment dummy × Performance-avoidance goal orientation-pre	–0.21	
Peer-assessment dummy × Performance-approach goal orientation-pre	0.51*	
Peer-assessment dummy × Performance-avoidance goal orientation-pre	−0.67**	

**p < 0.01. *p < 0.05.

Interaction between the peer-assessment dummy and performance-approach goal orientation-pre and the interaction between the peer-assessment dummy and performance-avoidance goal orientation-pre were significant. Simple slope tests were conducted to examine the two interactions that were significant. The regression lines of peer-assessment to the growth of learning goal orientation were calculated at the average of performance-approach goal orientation-pre ± 1 SD and the average of performance-avoidance goal orientation-pre ± 1 SD.

**FIGURE 2:**
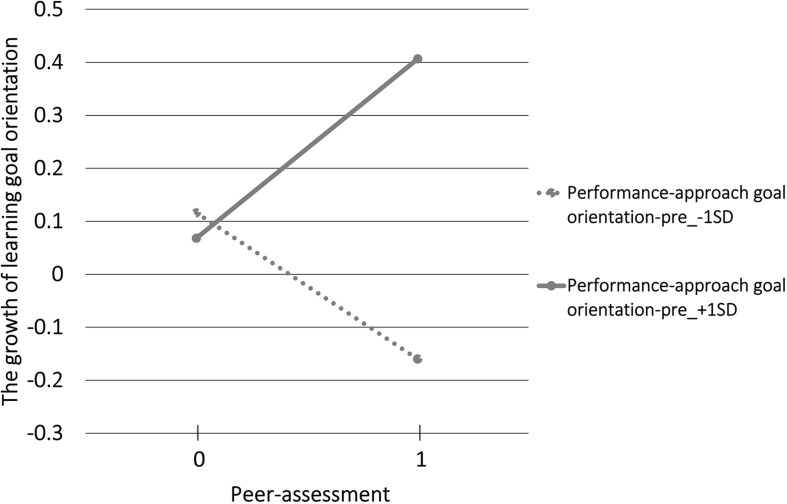
The regression line of peer-assessment to the growth of learning goal orientation with performance-approach goal orientation-pre as the moderating variable.

Figure 2 displays the regression line of peer-assessment to the growth of learning goal orientation with performance-approach goal orientation-pre as the moderating variable. The slope was marginally significantly negative (β = −0.44, *p* < 0.10) when performance-approach goal orientation-pre was low (−1 SD), and when performance-approach goal orientation-pre was high (+1 SD), the slope was significantly positive (β = 0.57, *p* < 0.05).

**FIGURE 3:**
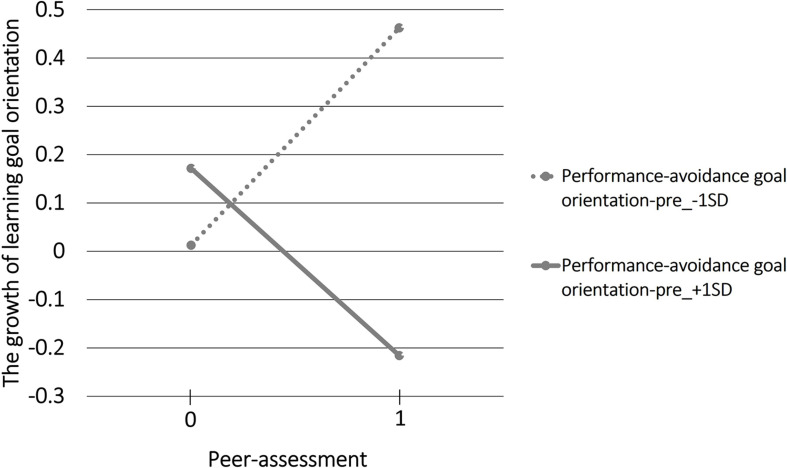
The regression line of peer-assessment to the growth of learning goal orientation with performance-avoidance goal orientation-pre as the moderating variable.

Figure 3 displays the regression line of peer-assessment to the growth of learning goal orientation with performance-avoidance goal orientation-pre as the moderating variable. When performance-avoidance goal orientation-pre was low (−1 SD), the slope was significantly positive (β = 0.75, *p* < 0.01), and when performance-avoidance goal orientation-pre was high (+1 SD), the slope was significantly negative (β = −0.62, *p* < 0.05).

As dummy variables representing the effect of using rubrics created by the students themselves, we assigned 1 to the participants who used a rubric created by the students themselves and 0 to the other participants (rubric construction dummy). As a result of multiple regression analysis with the growth of learning goal orientation as the dependent variable and the rubric construction dummy as the independent variable, no significant main effect was shown (β = −0.14, *n.s.*). In addition, neither the rubric construction dummy × self-assessment dummy interaction term nor the rubric construction dummy × peer-assessment dummy interaction term was significant, and no interaction effect was confirmed (β = −0.17, *n.s.*, β = 0.17, *n.s.*). From these results, it was judged that there was no effect of assessing using a rubric created by the students themselves.

## Discussion

### Effect of Peer-Assessment

The interaction between the peer-assessment dummy and performance-approach goal orientation-pre and the interaction between the peer-assessment dummy and performance-avoidance goal orientation-pre showed significant effects on the growth of learning goal orientation. These results suggest that the effect of peer-assessment on the growth of learning goal orientation may change depending on the initial performance goal orientation.

The effect of an intervention related to assessment methods on changes in learning goal orientation was not seen in Geitz et al. (2015), possibly because the effect of the intervention depends on students’ initial performance goal orientation. We think it is not enough to analyze the change in the rating values of learning goal orientation before and after the intervention; rather, it is necessary to analyze the influence of students’ initial performance goal orientation to observe the effect of the intervention.

We showed that peer-assessment has a positive effect on the growth of learning goal orientation when students’ initial performance-approach goal orientation is high. In contrast, peer-assessment has a negative effect on the growth of learning goal orientation when students’ initial performance-avoidance goal orientation is low.

Students who have high performance-approach goal orientation and high performance-avoidance goal orientation are different in how they accept assessments from others. Consequently, the opposite peer-assessment effect was seen.

Students who have a high performance-approach goal orientation tend to look positively at their own positive aspects and positively recognize them (Elliot and Church, 1997). When performing peer-assessment, such students will focus on positive assessments by other students. It is assumed that their positive recognition of assessment by other students may have led to increased motivation toward learning and active involvement in learning when performing peer-assessment. Moreover, students who have a high performance-approach goal orientation tend to adopt a deep learning approach (Liem et al., 2008). Such students will deeply interpret the contents of the assessments from other students and improve their skills. Subsequently, learning goal orientation increased.

Students who have high performance-avoidance goal orientation tend to be anxious about being assessed (Elliot and McGregor, 1999) and recognize their own negative information (Elliot and Church, 1997). It is assumed that such students felt uneasy about being assessed by other students and focused on their negative aspects. This may lead to a decrease in learning motivation. In addition, students with high performance-avoidance goal orientation tend to adopt a surface learning approach (Liem et al., 2008). It is presumed that such students made a shallow interpretation focusing on “whether the assessment from other students is high or low.” Owing to these factors, learning goal orientation decreased.

Another possibility is that students who have high performance-approach goal orientation and high performance-avoidance goal orientation may have different interpretations of the goals emphasized in this class. Students’ goal orientation is influenced by the goals emphasized in the class (Roeser et al., 2002); this encourages students’ individual learning goal orientations (Roeser et al., 1996; Anderman and Anderman, 1999).

We speculate that students who have high performance-approach goal orientation essentially understood the meaning of peer-assessment and regarded peer-assessment as a mechanism for improving their skills. This may lead them to recognize that the class focused on improving the learning process and skills, that is, learning goals. Subsequently, the learning goal orientation of such students increased.

We presumed that students who have high performance-avoidance goal orientation perceive peer-assessment superficially and see it merely as their performance being assessed by others. They may perceive the focus of this class as not being to improve their learning process or skills but to improve their performance. Subsequently, individual students’ learning goal orientations decreased.

### Effect of Self-Assessment

The main effect of the self-assessment dummy was not significant, and no self-assessment effect was observed. This may be because students were unable to make effective self-assessments, and hence, doing these did not provide an opportunity to improve their skills. It is difficult to assess yourself objectively over a short space of time and develop strategies to improve your learning skills. Hattie and Timperley (2007) stated that metacognitive skills need to be acquired for effective self-assessment. Seo et al. (2018) have shown that it is difficult to encourage students to use voluntary metacognitive strategies and that additional teacher encouragement and support are required. In this exercise, the teachers did not provide additional encouragement or support to students in the self-assessment group. From the above, we consider that the students in the self-assessment group could not perform self-assessment effectively, and the learning experience of self-assessment did not lead to the improvement of their skills and consequently did not constitute an opportunity to stimulate their learning goal orientation.

None of the effects of the interaction between the self-assessment dummy and performance-approach goal orientation-pre, and the interaction between self-assessment and performance-avoidance goal orientation-pre were significant. Self-assessment is a static learning activity that individual students complete by reviewing their own performance and organizing knowledge related to what they have learned. Performance-approach goal orientation and performance-avoidance goal orientation are orientations related to assessment by others and are not related to self-assessment, which is an individual activity; therefore, there was no moderating effect of performance-approach goal orientation and performance-avoidance goal orientation.

### Limitations and Prospects

The effects of learning are influenced by students, learning environments, teachers, and their interactions (Manalo et al., 2018). The results obtained in this study may change depending on how the learning environment is designed and how teachers are involved.

In this study, the effects of self-assessment were not observed, but this does not mean that there is no effect of such. As mentioned earlier, students need to acquire metacognitive skills for effective self-assessment (Hattie and Timperley, 2007). It is possible that self-assessment could be effective by incorporating training to acquire metacognitive skills as a learning environment design. Fong et al. (2018) stated that it is important for the receiver of the assessment to trust the giver to make an effective assessment. In our scenario, the partners of peer-assessment were just classmates, and it is possible that a sufficient relationship of trust could not be built over 5 weeks. If students who perform peer-assessment were perceived as reliable partners, it is possible that a higher peer-assessment effect could be obtained.

Regarding how teachers are involved, Manalo et al. (2018) stated that teacher involvement is necessary to increase students’ spontaneity and learning effectiveness. However, in this instance, the teachers did not actively talk to the students or guide them while the latter were conducting self-assessment and peer-assessment.

We have mentioned some points to be improved on how to design the learning environment and how to involve teachers; it is necessary to consider effective self-assessment and peer-assessment methods and to empirically examine the effect on the growth of learning goal orientation as important developments in the future.

### Educational Suggestions

Peer-assessment is considered effective in increasing students’ autonomy and motivation (Hiltz and Wellman, 1997; Brindley and Scoffield, 1998; Pope, 2001) and is often adopted in educational practice. This study suggests that peer-assessment does not positively affect all students but that the effects differ depending on their initial performance goal orientation. Moreover, it is effective for students who have high performance-approach goal orientation or low performance-avoidance goal orientation. It is oppositely effective for students who have high performance-avoidance goal orientation or low performance-approach goal orientation. Teachers should understand student profiles from the perspective of performance goal orientation and decide whether to incorporate peer-assessment into the course design.

## Conclusion

This study examined the effects of self- and peer-assessment on the growth of learning goal orientation, focusing on the moderating effects of the initial performance-approach goal orientation and performance-avoidance goal orientation. Therefore, the following suggestions were made: (1) the effect of peer-assessment on the growth of learning goal orientation may change depending on the initial performance-approach and performance-avoidance goal orientations; (2) peer-assessment would be effective in increasing learning goal orientation for students who have high performance-approach goal orientation or low performance-avoidance goal orientation; and (3) peer-assessment would be counterproductive in increasing learning goal orientation for students who have low performance-approach goal orientation or high performance-avoidance goal orientation.

This study provided basic knowledge regarding increasing students’ learning goal orientation, which has not been provided to date. Moreover, this study is significant to educational practice. It was able to reveal that the effect of peer-assessment on the growth of learning goal orientation may change depending on the initial performance goal orientation.

## Data Availability Statement

The raw data supporting the conclusions of this article will be made available by the authors, without undue reservation.

## Ethics Statement

The studies involving human participants were reviewed and approved by School of Human Science and Environment, University of Hyogo. The patients/participants provided their written informed consent to participate in this study.

## Author Contributions

Both authors contributed to the design and implementation of the research, analysis of the results, and writing of the manuscript.

## Conflict of Interest

The authors declare that the research was conducted in the absence of any commercial or financial relationships that could be construed as a potential conflict of interest.

## Publisher’s Note

All claims expressed in this article are solely those of the authors and do not necessarily represent those of their affiliated organizations, or those of the publisher, the editors and the reviewers. Any product that may be evaluated in this article, or claim that may be made by its manufacturer, is not guaranteed or endorsed by the publisher.
